# Risk-Based Triage Using Cytology and HPV Genotyping to Reduce Unnecessary Colposcopy: A Real-World Cross-Sectional Study

**DOI:** 10.3390/biomedicines14061224

**Published:** 2026-05-28

**Authors:** Sait Erbey, Mehmet Alican Sapmaz, Murat Polat, Ömer Osman Eroğlu, Çağanay Soysal

**Affiliations:** Department of Obstetrics and Gynecology, Ankara Etlik City Hospital, 06170 Ankara, Turkey; dr.alicansapmaz@hotmail.com (M.A.S.); dr.muratpolat@hotmail.com (M.P.); omerosmaneroglu@gmail.com (Ö.O.E.); cagnay@hotmail.com (Ç.S.)

**Keywords:** cervical cancer screening, HPV genotyping, colposcopy, risk-based triage, CIN3+, human papillomavirus, co-testing

## Abstract

**Background and Objectives:** Despite the widespread adoption of HPV-based cervical cancer screening, the optimal triage strategy for women with low-grade cytological abnormalities and non-16/18 high-risk HPV (hrHPV) types remains debated. This study evaluated the impact of ASCCP risk-based triage strategies on colposcopy referral and biopsy outcomes in a large tertiary care center. **Methods:** This retrospective cross-sectional study included 2748 sexually active women aged 30–65 years who underwent colposcopy at Ankara Etlik City Hospital (January 2023–June 2025). Of these, 1932 met ASCCP criteria for cervical biopsy. Cytology results, HPV genotypes (16, 18, and other hrHPV types), and histopathological findings were analyzed. CIN3+ (CIN3, adenocarcinoma in situ, or invasive carcinoma) was the primary outcome. Multivariable logistic regression identified independent predictors, with model fit assessed by Nagelkerke R^2^ and the Hosmer–Lemeshow test. **Results:** The mean age was 42.8 ± 8.1 years. The overall CIN3+ prevalence was 15.9% (308/1932). HSIL cytology was the strongest independent predictor of CIN3+ (adjusted OR 22.41, 95% CI: 11.28–44.52). HPV16/18 combined with HSIL or ASC-H cytology conferred the highest risk (adjusted OR 17.88–21.67). Women with ASC-US or LSIL cytology and non-16/18 hrHPV types had CIN3+ rates below 10%. Irregular screening history was also an independent predictor (adjusted OR 1.38). A risk-based triage approach suggested a potential reduction of approximately 29.7% in colposcopy utilization. However, this estimate applies exclusively to the biopsied subgroup and does not account for potentially undetected lesions in the 816 non-biopsied women enrolled in surveillance follow-up. **Conclusions:** HSIL cytology and HPV16/18 positivity represent the highest-risk profile for CIN3+ and should remain primary indications for colposcopy. Conversely, women with ASC-US or LSIL cytology and non-16/18 hrHPV types may be candidates for surveillance-based co-testing rather than immediate colposcopy, potentially enabling a resource-efficient reduction in unnecessary procedures within the biopsied cohort studied. Prospective validation in broader colposcopy-referred populations is needed before generalizing these findings to primary screening settings.

## 1. Introduction

Cervical cancer remains a major global health burden, ranking as the fourth most common malignancy among women worldwide, with an estimated 604,000 new cases and 342,000 deaths in 2020 [1]. Given its well-established progression through identifiable precancerous stages, invasive disease is largely preventable through timely detection and treatment of cervical intraepithelial neoplasia (CIN). Accordingly, population-based screening programs constitute the cornerstone of prevention, with many high-income countries transitioning toward HPV-based primary screening and risk-stratified management strategies [2,3,4].

Persistent infection with high-risk human papillomavirus (hrHPV) represents the central etiological driver of cervical carcinogenesis [5]. Therefore, optimizing triage strategies in HPV-positive populations is not merely a clinical management issue but a critical component of infection-driven cancer prevention. In this context, risk-based approaches that integrate viral genotype with host cytological response may provide a biologically grounded framework for improving both the efficiency and precision of cervical cancer control.

In Turkey, cervical cancer accounts for approximately 4–5 cases per 100,000 women, ranking among the ten most common malignancies in females [6]. High-grade squamous intraepithelial lesions (HSIL) are most closely associated with persistent high-risk HPV (hrHPV) infection [7]. HPV DNA testing offers higher sensitivity than Pap smear cytology for primary cervical cancer screening [8]. The national screening program was expanded in 2014 to incorporate co-testing: women aged 30–65 undergo liquid-based Pap cytology and HPV DNA testing concurrently. If both are negative, repeat testing is performed at five-year intervals. When HPV DNA is positive, genotyping is required, and colposcopy may be indicated depending on the cytological result and HPV genotype detected.

The 2019 Risk-Based Management Consensus Guidelines of the American Society for Colposcopy and Cervical Pathology (ASCCP) integrate current and prior cytology results, HPV status, and genotype information to stratify patients according to immediate and future risk thresholds for CIN3+ [9]. While this framework improves sensitivity for high-grade disease, it also leads to a substantial proportion of women being referred for colposcopy despite low clinical risk. Colposcopy requires trained operators, and when performed without sufficient expertise, it may lead to misguidance, elevated costs, and false-negative results [10]. Beyond procedural limitations, unnecessary colposcopy is associated with significant patient-level harms, including anxiety, pain, bleeding, and infection risk [11,12]. Women who undergo subsequent cervical excisional procedures face elevated obstetric risks, including preterm birth and low birth weight [13].

Despite these concerns, the optimal triage strategy for women with low-grade cytological abnormalities and non-16/18 hrHPV types remains an area of active clinical debate. Immediate colposcopy for all hrHPV-positive women may generate a considerable number of unnecessary procedures without meaningfully improving high-grade lesion detection. Evaluating real-world data from large patient cohorts is therefore essential to determine whether a more selective, risk-based approach to colposcopy referral can improve the benefit–harm balance of cervical cancer screening programs.

The present study aimed to evaluate the real-world performance of ASCCP risk-based triage strategies in a large tertiary referral cohort and to quantify the incremental value of combining cytology and HPV genotyping for predicting CIN3+. In addition, we sought to identify clinically actionable risk strata that could support more selective and resource-efficient colposcopy referral practices.

## 2. Materials and Methods

### 2.1. Ethics Statement

This study was approved by the Ankara Etlik City Hospital No. 1 Scientific Research Evaluation and Ethics Committee (Decision No: AEŞH-BADEK1-2025-058; 7 May 2025). All procedures were conducted in accordance with the Declaration of Helsinki. The requirement for informed consent was waived due to the retrospective study design and the use of fully anonymized data.

### 2.2. Study Design and Population

This retrospective cross-sectional study included 2748 sexually active women aged 30–65 years who underwent colposcopic examination at a tertiary referral center between January 2023 and June 2025.

Data on HPV DNA test results (HPV16, HPV18, and other high-risk HPV types), liquid-based cytology, colposcopy indications, and histopathological outcomes were extracted from electronic medical records. Exclusion criteria included absence of sexual activity, age outside the 30–65-year range, active systemic disease, prior diagnosis or treatment of cervical cancer or CIN, and incomplete clinical data. ‘Regularly screened’ was defined as at least one documented HPV/cytology co-test within the five years preceding referral (any missed intervals not penalized); all others, including never-screened women, were classified as ‘irregular/unscreened’.

### 2.3. Colposcopy and Biopsy Protocol

HPV DNA testing (Cobas HPV Test, Roche Molecular Systems, Pleasanton, CA, USA; providing separate genotyping for HPV16, HPV18, and pooled other hrHPV types) and liquid-based cytology (ThinPrep, Hologic, Marlborough, MA, USA) were performed per Turkish Ministry of Health protocol. Colposcopy and biopsy decisions were guided by the 2019 ASCCP Risk-Based Management Consensus Guidelines. Among 2748 women undergoing colposcopy, 1932 (70.3%) met predefined criteria for cervical biopsy. The remaining 816 women (29.7%) did not undergo biopsy, as they fulfilled all predefined low-risk criteria: (i) normal colposcopic findings with a fully visible squamocolumnar junction and absence of acetowhite changes, metaplasia, or abnormal vascular patterns; (ii) cytology less severe than HSIL; and (iii) negative HPV16/18 status. Per ASCCP guidelines, random untargeted biopsy is not recommended in this constellation, given a negligible incremental CIN3+ detection yield. These women were enrolled in a 12–24-month repeat co-testing surveillance protocol. In accordance with current guidelines, random biopsies were not performed in this subgroup, although the possibility of missed occult high-grade lesions cannot be entirely excluded.

Among biopsied women, two to four targeted biopsies were obtained from all acetowhite areas. HPV16/18 co-infection was identified in 143 patients (5.2%) and was classified according to the highest oncogenic-risk genotype (HPV18 when both genotypes were present) for genotype-stratified analyses. The primary outcome was defined as CIN3+, encompassing CIN3, adenocarcinoma in situ (AIS), and invasive carcinoma.

### 2.4. Statistical Analysis

Categorical variables are presented as absolute frequencies and percentages. Intergroup comparisons used the Chi-square test or Fisher’s exact test as appropriate. Bonferroni correction was applied for multiple pairwise comparisons; corrected *p*-values are reported throughout. Relative risks (RR) and odds ratios (OR) with 95% confidence intervals (CI) were calculated from 2 × 2 contingency tables for pre-specified comparisons. All point estimates are reported to two decimal places.

To identify independent predictors of CIN3+, a multivariable logistic regression model was constructed with CIN3+ as the dependent variable. Candidate covariates were first evaluated by univariate logistic regression; those with *p* < 0.10 were entered into the multivariable model. These included cytology category (reference: ASC-US), HPV16/18 genotype combined with cytology grade, age group, BMI category, parity, and screening regularity. HPV genotype was entered as two binary variables: (1) HPV16/18 co-occurring with HSIL or ASC-H cytology, and (2) HPV16/18 in the absence of high-grade cytology (reference: HPV Negative, n = 10). Given the very small HPV-negative reference group (n = 10), OR estimates for HPV16/18 alone versus HPV Negative should be interpreted with caution as they are statistically unstable; these estimates are retained in the model for completeness but are not used to draw clinical conclusions. Model fit was assessed using the Hosmer–Lemeshow goodness-of-fit test (acceptable fit: *p* > 0.05) and the Nagelkerke R^2^. Results are presented as adjusted ORs with 95% CIs and corresponding *p*-values. Analyses were performed on complete-case data using SPSS version 26.0 (IBM Corp., Armonk, NY, USA). Statistical significance was defined as *p* < 0.05 (two-tailed). A post hoc sensitivity analysis was additionally performed in which the HPV-negative reference group was pooled with the HPV Other+ subgroup to form a combined non-16/18 reference (n = 75), providing more stable OR estimates for the HPV16+ alone and HPV18+ alone comparisons.

## 3. Results

The mean age of the 2748 included women was 42.8 ± 8.1 years. Most participants were multiparous (91.0%), with a median parity of 2 (range, 0–6). The mean BMI was 26.2 ± 4.3 kg/m^2^; 31.5% of participants were overweight, and 18.2% were obese. A total of 68.3% of participants had undergone at least one cesarean section. Regarding screening history, 58.9% had undergone regular co-testing within the past five years, whereas 41.1% had irregular or no prior screening history (Table 1).

Combined evaluation of cytology and HPV genotype identified HSIL with HPV16/18 positivity as the highest-risk subgroup for CIN3+. In contrast, ASC-US or LSIL cytology combined with non-16/18 hrHPV types was associated with CIN3+ rates below 10% and predominantly benign histopathology (Table 2).

A clinically meaningful gradient in CIN3+ risk was observed across combined cytology and HPV genotype categories. Women with HSIL or ASC-H cytology in the presence of HPV16/18 demonstrated very high CIN3+ risks ranging from 63% to 84%. In contrast, women with ASC-US or LSIL cytology and non-16/18 hrHPV types had substantially lower risks, approximately 5–8%. Intermediate risk levels were observed in HPV16/18-positive women with low-grade cytology. This stratified risk distribution highlights the importance of integrating cytology and HPV genotyping for more precise risk-based clinical decision-making (Table 3).

When stratified by cytology alone, CIN3+ prevalence increased progressively with cytological severity. HSIL yielded the highest rate (74.5%), followed by ASC-H (58.1%). AGC carried a 15.0% CIN3+ rate (3/20), warranting clinical attention despite the small subgroup size. Low-grade categories (ASC-US, LSIL) had substantially lower rates. When further stratified by HPV genotype, HPV18-positive women demonstrated the highest overall CIN3+ prevalence (18.3%), modestly but significantly higher than HPV16-positive women (14.5%; *p* = 0.036). Women with non-16/18 hrHPV types or HPV-negative status had markedly lower rates. These data are summarised in Table 4. To address the possibility that this difference was driven by differential cytology distribution between genotypes, a post hoc Mantel–Haenszel analysis stratified by cytology category was performed. The cytology distribution was virtually identical between groups (high-grade cytology [HSIL + ASC-H]: 14.5% in HPV16+ vs. 15.4% in HPV18+), and HPV18 retained numerically higher CIN3+ rates within each cytology stratum (Table 5). The cytology-adjusted Mantel–Haenszel OR (HPV18 vs. HPV16) was 1.44 (95% CI 1.05–1.98), similar to the crude OR of 1.31 (95% CI 1.02–1.68), with both confidence intervals excluding unity, indicating that the observed difference is not explained by cytology-grade confounding.

Comparative risk analysis showed that HSIL was associated with a 7.37-fold increased risk of CIN3+ compared with ASC-US (RR 7.37, 95% CI: 4.63–11.72; OR 25.99, 95% CI: 13.36–50.54), while ASC-H was associated with a 5.74-fold increased risk (RR 5.74, 95% CI: 3.61–9.14; OR 12.31, 95% CI: 6.95–21.81). RR estimates for HPV16+ and HPV18+ versus HPV-negative status were modest, with confidence intervals crossing unity, reflecting the small HPV-negative subgroup (n = 10) (Table 6).

Univariate logistic regression was performed for all candidate covariates to inform variable selection for the multivariable model. Key univariate associations are summarized in Table 7. Variables demonstrating *p* < 0.10 on univariate analysis were carried forward into the multivariable model.

In multivariable logistic regression (Nagelkerke R^2^ = 0.41; Hosmer–Lemeshow χ^2^ = 6.83, *p* = 0.557), HSIL cytology emerged as the strongest independent predictor of CIN3+ (adjusted OR 22.41, 95% CI: 11.28–44.52; *p* < 0.001). HPV16/18 combined with HSIL or ASC-H cytology also conferred high independent risk (adjusted ORs 17.88–21.67). Irregular screening history was independently associated with CIN3+ (adjusted OR 1.38, 95% CI: 1.07–1.77, *p* = 0.013). HPV genotype alone, in the absence of high-grade cytology, was not independently associated with CIN3+. Age and BMI were not significantly associated with CIN3+ in the multivariable model (Table 8). In a post hoc sensitivity analysis using a pooled non-16/18 reference (HPV-Negative + HPV Other+, n = 75), OR estimates for HPV16+ alone (OR 1.43, 95% CI 0.67–3.03) and HPV18+ alone (OR 1.87, 95% CI 0.88–3.98) yielded markedly narrower confidence intervals than the primary analysis (HPV16+ OR 1.53, 95% CI 0.19–12.18; HPV18+ OR 2.01, 95% CI 0.25–15.99). Although the pooled-reference estimates remained non-significant, the direction of effect was preserved, supporting the conclusion that HPV genotype alone, in the absence of high-grade cytology, is not an independent predictor of CIN3+.

Application of a risk-based triage strategy demonstrated a substantial potential to reduce unnecessary colposcopy procedures. Under the current approach, all 2748 women underwent colposcopic evaluation, whereas a risk-based strategy restricting immediate colposcopy to higher-risk groups would have limited procedures to 1932 women. This represents a potential reduction of approximately 29.7% in colposcopy utilization without observed loss of CIN3+ detection within the biopsied cohort, acknowledging that undetected lesions in the non-biopsied group cannot be excluded. This reduction refers to colposcopic examinations rather than biopsies—under the proposed algorithm, the 816 low-risk women would have been deferred to surveillance co-testing without colposcopy—and assumes no loss of CIN3+ detection within the biopsied subgroup; the true miss rate cannot be determined from this retrospective design (Table 9).

**Figure 1:**
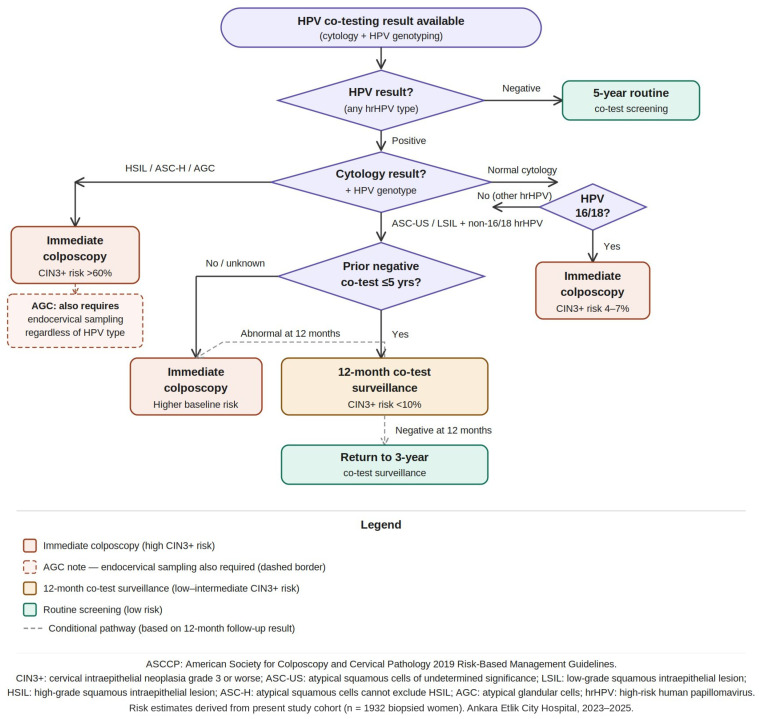
Proposed risk-stratified colposcopy referral algorithm. Immediate colposcopy is indicated for women with HSIL, ASC-H, AGC, or HPV16/18 positivity regardless of cytology (red boxes). Women with ASC-US or LSIL cytology and non-16/18 hrHPV types without a prior negative co-test within 5 years are also referred to colposcopy; those with a documented prior negative result are managed with 12-month co-test surveillance (amber box). Women negative at 12 months return to 3-year surveillance (green box). AGC cases require colposcopy with endocervical sampling regardless of HPV genotype (dashed border). AGC: atypical glandular cells; ASC-H: atypical squamous cells, cannot exclude HSIL; ASC-US: atypical squamous cells of undetermined significance; CIN3+: cervical intraepithelial neoplasia grade 3 or worse; hrHPV: high-risk human papillomavirus; HSIL: high-grade squamous intraepithelial lesion; LSIL: low-grade squamous intraepithelial lesion.

As shown in Table 9, high-grade cytology combined with HPV16/18 positivity yielded positive predictive values exceeding 60% in ASC-H and 79–84% in HSIL categories, while low-grade cytology with non-16/18 hrHPV types showed values below 10%. Based on these stratified risk estimates, a clinical decision algorithm integrating HPV genotype, cytology grade, and prior screening history was constructed to operationalize risk-based colposcopy referral (Figure 1).

## 4. Discussion

The principal finding of this study is that HSIL or ASC-H cytology combined with HPV16/18 positivity represents the highest-risk profile for CIN3+, while ASC-US or LSIL cytology with non-16/18 hrHPV types carried rates below 10%. HPV genotype alone, in the absence of high-grade cytology, was not independently associated with CIN3+ in the adjusted model. These findings support a shift toward risk-adapted colposcopy management in which cytology grade and HPV genotype are assessed jointly—HPV genotype reflecting viral oncogenic potential and cytological abnormalities reflecting the host epithelial response—providing a biologically coherent framework for stratification.

These findings are consistent with, and further extend, evidence from large international screening populations. Kaljouw et al. demonstrated, in a modelling study of the Dutch screening program, that incorporating HPV16/18 genotyping and increasing cytological thresholds for non-16/18 hrHPV types can significantly reduce unnecessary colposcopy referrals without compromising cervical cancer incidence or mortality outcomes [14]. In a large Korean screening cohort, Cho et al. showed that HPV16/18 genotyping provides substantial risk stratification for CIN3+, even among women with negative cytology, thereby supporting its integration into primary triage strategies [15]. The ESTAMPA multicentric study, conducted across diverse low- and middle-income settings, similarly showed that genotype-guided HPV triage and high-quality colposcopy maintain high detection rates of cervical precancer across heterogeneous populations [16]. Data from high-altitude populations in China further support the role of hrHPV genotype distribution as a key determinant of CIN3+ prevalence across diverse geographic and demographic settings [17]. Taken together, the convergence of evidence from modelling studies, prospective cohorts, and multicentric trials strengthens the generalizability of our real-world findings.

A noteworthy finding in our dataset was the slightly but statistically significantly higher CIN3+ prevalence observed in HPV18-positive women (18.3%) compared with HPV16-positive women (14.5%; *p* = 0.036). To examine whether this difference could be attributable to differential cytology distribution between genotype groups, a post hoc Mantel–Haenszel analysis stratified by cytology category was conducted (see Section 3). The cytology distribution was virtually identical between HPV16- and HPV18-positive women, and the cytology-adjusted Mantel–Haenszel OR (1.44) was similar to the crude OR (1.31), indicating that the observed difference is not explained by cytology-grade confounding. The finding is biologically plausible: HPV18 has been shown to be disproportionately associated with adenocarcinoma and adenocarcinoma in situ (AIS)—pathologies that may not manifest as overt acetowhite lesions at colposcopy and are therefore at heightened risk of being missed or underdiagnosed [18]. This pattern likely reflects the disproportionate association of HPV18 with glandular lesions, which may not manifest as overt acetowhite changes at colposcopy, underscoring the value of endocervical sampling in HPV18-positive cases even when ectocervical findings appear unremarkable.

An additional clinically relevant finding is the non-negligible CIN3+ prevalence observed among women with normal cytology and HPV16/18 positivity, reaching 4.3% for HPV16+ (32/745) and 7.2% for HPV18+ (40/557) cases. Although these rates are lower than those observed in the high-grade cytology subgroups, they represent clinically relevant absolute risks. The 2019 ASCCP Risk-Based Management Consensus Guidelines define an immediate CIN3+ risk threshold of ≥4% for colposcopy referral [9], indicating that both HPV16+ and HPV18+ normal-cytology groups in our cohort exceeded this clinically relevant threshold. Taken together, these CIN3+ rates provide direct real-world empirical validation, from a middle-income country tertiary care setting, of the guideline recommendation to refer HPV16/18-positive women to colposcopy regardless of cytological result. Routine deferral of colposcopy on the basis of normal cytology alone is not supported by these findings in HPV16/18-positive women.

These findings confirm that cytological severity remains the dominant risk determinant regardless of HPV genotype, and that HSIL warrants colposcopy irrespective of HPV type. Notably, LSIL also emerged as a statistically significant independent predictor of CIN3+ in the multivariable model (adjusted OR 2.18, 95% CI: 1.05–4.52; *p* = 0.036), a finding that merits attention. While this association is modest compared with HSIL and ASC-H, it suggests that LSIL should not be unconditionally regarded as low risk in the context of concurrent hrHPV infection, and reinforces the importance of combined cytology–genotype assessment rather than reliance on cytology grade alone.

The AGC subgroup, although small (n = 20), demonstrated a CIN3+ prevalence of 15.0% (3/20), consistent with established evidence that atypical glandular cells are associated with a disproportionately elevated risk of both squamous and glandular high-grade lesions. Our findings support direct colposcopy referral with endocervical sampling for all AGC cases regardless of HPV genotype, in line with ASCCP and ESGO recommendations, acknowledging that the small subgroup size limits statistical inference.

For women with non-16/18 hrHPV types and low-grade cytology, deferred co-testing appears to be justified on both clinical and health-economic grounds. A Singapore-based health economic model showed that p16/Ki-67 dual-stained cytology, used as a reflex triage tool in this group, is more cost-effective than immediate colposcopy, while safely extending follow-up intervals in dual-stain-negative women without compromising high-grade lesion detection [19]. A Norwegian population-based study similarly showed that retesting, rather than immediate colposcopy, for women with non-16/18 hrHPV types and low-grade cytology more effectively balances the benefits and harms of screening compared with direct referral [20]. The cost-effectiveness of HPV-based screening strategies incorporating genotype stratification has also been demonstrated in the Dutch healthcare context, where such approaches were shown to be cost-effective at standard willingness-to-pay thresholds [21]. Together, these findings support a more selective, risk-based approach to colposcopy referral that optimizes both clinical outcomes and healthcare resource utilization.

The independent association between irregular screening history and CIN3+ (adjusted OR 1.38, 95% CI: 1.07–1.77) represents a clinically and public health–relevant finding beyond primary virological and cytological predictors. Population-based evidence consistently shows that the majority of cervical cancer cases occur in women who have been unscreened or inadequately screened in the years preceding diagnosis [22]. Women presenting to colposcopy units with inadequate prior screening histories are, by definition, at a more advanced stage in the natural history of HPV infection and CIN progression. This selection bias likely explains the higher CIN3+ burden in the irregularly screened group, and has direct implications for clinical practice: women without prior regular co-testing should be considered a distinct higher-risk group warranting more intensive follow-up. These findings reinforce the public health priority of improving screening access and patient recall in settings like Turkey, where 41.1% of our cohort had no regular prior screening.

This study provides real-world evidence from a middle-income country context that has been underrepresented in the international HPV triage literature. Turkey’s national co-testing program, introduced in 2014, represents a substantial investment in population-level cervical cancer prevention infrastructure. However, the capacity and expertise required for colposcopic evaluation of all hrHPV-positive referrals remain unevenly distributed across the healthcare system. Our findings suggest that a selective, risk-stratified colposcopy referral strategy, focused on women with high-grade cytology or HPV16/18 positivity, could substantially reduce colposcopy burden, pending prospective confirmation that high-grade lesion detection is preserved in the deferred subgroup. This approach would allow colposcopy resources to be concentrated on the highest-risk patients, thereby improving quality of care and reducing waiting times in capacity-limited settings. The generalizability of this approach to other middle-income countries with similar co-testing infrastructure warrants further prospective evaluation.

Two recent studies from Turkish tertiary centers are particularly relevant to contextualizing our findings. Kaya Terzi and Yulek (2024) reported sensitivity and specificity data for co-testing in a Turkish cohort (n = 225) [23]. Similarly, Yalcin et al. (2026) evaluated colposcopy outcomes in 2682 women referred for hrHPV positivity and/or abnormal cytology at a tertiary center, confirming that HPV16 and HPV18 represent the highest-risk groups for CIN2+ lesion development [24]. The present study substantially extends this body of literature. Compared with prior studies, it includes a larger sample size (n = 2748), applies the ASCCP 2019 risk-based management framework [9], and uses CIN3+ as the primary endpoint, in alignment with current guideline thresholds for treatment. In addition, it provides multivariable risk estimates with formal model-fit statistics and identifies clinically relevant risk stratification patterns, including the observed HPV18 > HPV16 signal, the elevated risk associated with HSIL in non-16/18 hrHPV cases, and the independent contribution of irregular screening history. These findings may have important implications for colposcopy triage protocols in referral settings, particularly in reducing unnecessary procedures and optimizing resource utilization; whether they apply to primary population-based screening programs requires prospective validation.

### 4.1. Clinical Implications

Before considering implementation, it must be emphasized that the risk estimates and thresholds derived in this study originate from a colposcopy-referred population, in which disease prevalence is substantially higher than in unselected primary screening cohorts; the proposed algorithm, therefore, should not be applied to primary screening settings without prospective validation in that context. Incorporating HPV16/18 genotyping into cytology-based triage enables a more selective and risk-proportionate approach to colposcopy referral. In this single-center cohort, approximately 29.7% of women referred for colposcopy met predefined low-risk criteria and did not undergo biopsy; whether this proportion is generalizable to other settings depends on local HPV prevalence, cytology thresholds, and referral patterns. Subject to that caveat, a practical risk-stratified triage algorithm based on our findings would recommend: (a) immediate colposcopy for women with HSIL, ASC-H, AGC, or HPV16/18 positivity, regardless of cytology grade; (b) 12-month co-testing surveillance for women with ASC-US or LSIL cytology and non-16/18 hrHPV types; and (c) five-year repeat co-testing for women with normal cytology and negative HPV (Figure 1). This further differentiation is supported by the independent association of irregular screening history with CIN3+ in our cohort (adjusted OR 1.38; Table 8) and by the explicit incorporation of prior screening results into risk thresholds in the 2019 ASCCP guidelines [9]. Such an approach may reduce unnecessary colposcopic procedures, minimize patient anxiety and procedure-related complications, decrease healthcare costs, and preserve the sensitivity of screening programs for detecting clinically significant lesions, while remaining aligned with the risk-threshold principles of the ASCCP 2019 guidelines. To contextualise the potential miss rate of this deferral strategy, the POBASCAM trial reported a 5-year CIN3+ risk of only 0.2% in women with negative HPV and cytology [25], and the ASCUS-LSIL Triage Study showed a 2-year CIN3+ risk of 11–13% in women with ASC-US or LSIL and a negative or CIN1 colposcopy [26]. Applied to the 816 non-biopsied women in our cohort—all with normal colposcopy, cytology below HSIL, and absent HPV16/18—these benchmarks suggest a substantially lower CIN3+ risk than the biopsied group; however, a definitive miss-rate estimate requires prospective follow-up with mandatory histological ascertainment.

From a health system perspective, the implementation of such a risk-based triage approach may have substantial implications for resource allocation, particularly in settings with limited colposcopy capacity. By reducing unnecessary referrals while maintaining detection of high-grade lesions, this strategy may improve efficiency, shorten waiting times, and enhance the overall quality of cervical cancer screening programs. These findings provide real-world validation of risk-based triage strategies and support their implementation in resource-constrained healthcare systems.

### 4.2. Limitations

This study has several important limitations. First, the retrospective cross-sectional design precluded prospective follow-up, and some variables were limited to those available in electronic records. Second, and most importantly, 816 women (29.7%) who underwent colposcopy did not meet criteria for biopsy and are not represented in the CIN3+ outcome analyses. The absence of biopsy data in this subgroup means that occult high-grade lesions cannot be excluded, and the reported efficiency of the risk-based triage strategy must be interpreted within this constraint: the claim that CIN3+ detection was preserved applies only to the biopsied cohort, not the full colposcopy population. This structural limitation should be addressed in future prospective studies with complete histological follow-up. As a corollary of this limitation, women with normal cytology and non-16/18 hrHPV positivity were not represented in the biopsied cohort, and the CIN3+ risk in this specific subgroup cannot be directly estimated from our data; management recommendations for this group therefore rely on guideline-based extrapolation rather than on cohort-specific evidence. Third, the colposcopy-referred population represents a high-risk clinical cohort; CIN3+ rates and triage performance metrics reported here should not be directly extrapolated to primary screening populations. Fourth, the single-center design may limit the generalizability of the findings to settings with different HPV prevalence profiles or referral thresholds. Fifth, important confounders, including smoking status, number of lifetime sexual partners, and history of prior CIN treatment, were not systematically available and therefore could not be included in the regression model. Sixth, HPV vaccination status was not available for analysis. Notably, Turkey launched a school-based national HPV vaccination program in 2023, overlapping with the study period (2023–2025), and the proportion of vaccinated women in the cohort is therefore unknown. As HPV vaccination reduces the prevalence of vaccine-targeted genotypes, including HPV16 and HPV18, the observed CIN3+ rate distributions may differ in future cohorts as vaccination coverage increases, and the findings should be interpreted in this transitional epidemiological context. Seventh, HPV co-infection was simplified through primary genotype assignment; future studies should more rigorously assess the impact of dual-genotype infections on CIN3+ risk. Finally, observer-dependent variability in colposcopic interpretation cannot be fully excluded, although all procedures were performed by experienced gynecologists in a high-volume referral center.

Key strengths of this study include the large sample size (n = 2748), providing robust statistical power; the integrated analysis of cytology, HPV genotyping, and histopathology in a real-world setting; and the use of multivariable analysis with model-fit statistics to identify independent predictors of CIN3+ beyond simple frequency comparisons.

## 5. Conclusions

In this large real-world cohort, integrating cytology grade with HPV genotype provided clinically actionable risk stratification for CIN3+. HSIL and HPV16/18 positivity identified the highest-risk women warranting immediate colposcopy, while ASC-US or LSIL with non-16/18 hrHPV types supported a surveillance-based approach. This risk-stratified strategy could substantially reduce unnecessary colposcopy within a colposcopy-referred cohort; however, its safety with respect to occult high-grade lesion detection in the deferred subgroup remains unproven and must be confirmed in prospective studies with complete histological follow-up of all referred women before application to primary screening settings.

## Figures and Tables

**Table 1:** Demographic and Clinical Characteristics of the Study Population (n = 2748).

Characteristic	Value (n = 2748)
Age, years (mean ± SD)	42.8 ± 8.1
Age groups, n (%)	
30–39 years	1012 (36.8%)
40–49 years	1189 (43.3%)
50–59 years	442 (16.1%)
≥60 years	105 (3.8%)
Parity, median (range)	2 (0–6)
Nulliparous	248 (9.0%)
Multiparous (≥1 delivery)	2500 (91.0%)
Mode of delivery	
Cesarean section (any)	1878 (68.3%)
Vaginal delivery only	624 (22.7%)
BMI, kg/m^2^ (mean ± SD)	26.2 ± 4.3
Normal (<25)	1382 (50.3%)
Overweight (25–29.9)	865 (31.5%)
Obese (≥30)	501 (18.2%)
Screening history (last 5 years)	
Regularly screened	1619 (58.9%)
Irregular/unscreened	1129 (41.1%)

BMI: body mass index; SD: standard deviation.

**Table 2:** Distribution of CIN3+ by HPV Type and Cytology (n = 1932 biopsied women).

HPV	Cytology	CIN3+ Positive (n)	CIN3+ Negative (n)	Total (n)	Ratio (CIN3+/n, 95% CI)
HPV16+	NORMAL	32	713	745	0.043 (0.031–0.060)
HPV16+	ASC-US	7	77	84	0.083 (0.041–0.162)
HPV16+	AGC	2	6	8	0.250 (0.071–0.591)
HPV16+	ASC-H	66	38	104	0.635 (0.539–0.721)
HPV16+	LSIL	7	55	62	0.113 (0.056–0.215)
HPV16+	HSIL	39	10	49	0.796 (0.664–0.885)
HPV18+	NORMAL	40	517	557	0.072 (0.053–0.096)
HPV18+	ASC-US	9	58	67	0.134 (0.072–0.236)
HPV18+	AGC	1	5	6	0.167 (0.030–0.564)
HPV18+	ASC-H	54	27	81	0.667 (0.559–0.760)
HPV18+	LSIL	7	44	51	0.137 (0.068–0.257)
HPV18+	HSIL	36	7	43	0.837 (0.700–0.919)
HPV Other+	ASC-US	1	16	17	0.059 (0.010–0.270)
HPV Other+	AGC	0	4	4	0.000 (0.000–0.490)
HPV Other+	ASC-H	2	19	21	0.095 (0.027–0.289)
HPV Other+	LSIL	1	12	13	0.077 (0.014–0.333)
HPV Other+	HSIL	3	7	10	0.300 (0.108–0.603)
HPV Negative	All categories ^†^	1	9	10	0.100 (0.018–0.404)

HPV: human papillomavirus; CIN3+: cervical intraepithelial neoplasia grade 3 or worse; CI: confidence interval. HPV16/18 co-infection was present in 143 women (5.2% of total cohort); these were classified under HPV18 for genotype-stratified analysis. ^†^ HPV-negative women who met biopsy criteria (n = 10 total across all cytology categories) are presented as a single consolidated row, as cytology-stratified analysis was not performed for this subgroup due to small cell sizes. No women with HPV Other+ genotype and normal cytology met predefined biopsy criteria (normal colposcopic findings, cytology below HSIL, and HPV16/18-negative status); this subgroup was therefore enrolled in the 12–24-month surveillance protocol and is absent from Table 2.

**Table 3:** Risk Stratification of CIN3+ Based on Combined Cytology and HPV Genotype.

Cytology	HPV Type	n	CIN3+ Risk (%)	95% CI	Clinical Risk Category
HSIL	HPV16	49	79.6%	(66.4–88.5)	Very high risk
HSIL	HPV18	43	83.7%	(70.0–91.9)	Very high risk
ASC-H	HPV16	104	63.5%	(53.9–72.1)	High risk
ASC-H	HPV18	81	66.7%	(55.9–76.0)	High risk
LSIL	HPV16	62	11.3%	(5.6–21.5)	Intermediate risk
LSIL	HPV18	51	13.7%	(6.8–25.7)	Intermediate risk
ASC-US	HPV16	84	8.3%	(4.1–16.2)	Low–intermediate risk
ASC-US	HPV18	67	13.4%	(7.2–23.6)	Intermediate risk
ASC-US/LSIL	non-16/18 hrHPV	30	5.9–7.7%	(see Table 2)	Low risk

HPV: human papillomavirus; ASC-H: atypical squamous cells, cannot exclude HSIL; ASC-US: atypical squamous cells of undetermined significance; HSIL: high-grade squamous intraepithelial lesion; LSIL: low-grade squamous intraepithelial lesion; CI: confidence interval. n = number of biopsied women in each stratum; 95% CI values are taken directly from Table 2. For ASC-US/LSIL combined with non-16/18 hrHPV, n reflects the pooled subgroup (ASC-US Other+ n = 17, LSIL Other+ n = 13); CI is shown as range (see Table 2 for stratum-specific values). Clinical risk categories were defined pragmatically based on observed CIN3+ proportions to reflect potential decision thresholds for colposcopy referral.

**Table 4:** CIN3+ Prevalence by Cytology Category and HPV Genotype (n = 1932 biopsied women).

Cytology	CIN3+ Positive (n)	CIN3+ Negative (n)	Total (n)	Ratio (CIN3+/n, 95% CI)
NORMAL	72	1230	1302	0.055 (0.044–0.069)
ASC-US	17	151	168	0.101 (0.064–0.156)
AGC	3	17	20	0.150 (0.052–0.360)
ASC-H	122	88	210	0.581 (0.513–0.646)
LSIL	15	111	126	0.119 (0.073–0.187)
HSIL	79	27	106	0.745 (0.655–0.819)
*HPV genotype*				
HPV16+	153	899	1052	0.145 (0.125–0.168)
HPV18+	147	658	805	0.183 (0.157–0.211)
HPV Other+	7	58	65	0.108 (0.053–0.206)
HPV Negative	1	9	10	0.100 (0.018–0.404)

AGC: atypical glandular cells; ASC-H: atypical squamous cells, cannot exclude HSIL; ASC-US: atypical squamous cells of undetermined significance; CI: confidence interval; HSIL: high-grade squamous intraepithelial lesion; LSIL: low-grade squamous intraepithelial lesion. All intergroup comparisons for cytology categories: *p* < 0.001 (Bonferroni-corrected Chi-square). *p* = 0.036 for HPV18+ vs. HPV16+ comparison (Bonferroni-corrected Chi-square).

**Table 5:** Cytology-Stratified Comparison of CIN3+ Risk: HPV18 vs. HPV16 (Post hoc Mantel–Haenszel Analysis).

Cytology	HPV16+ CIN3+/n (%)	HPV18+ CIN3+/n (%)	Stratum OR (95% CI)
NORMAL	32/745 (4.3%)	40/557 (7.2%)	1.72 (1.07–2.78)
ASC-US	7/84 (8.3%)	9/67 (13.4%)	1.71 (0.60–4.85)
LSIL	7/62 (11.3%)	7/51 (13.7%)	1.25 (0.41–3.83)
AGC	2/8 (25.0%)	1/6 (16.7%)	0.60 (0.04–8.73)
ASC-H	66/104 (63.5%)	54/81 (66.7%)	1.15 (0.63–2.12)
HSIL	39/49 (79.6%)	36/43 (83.7%)	1.32 (0.45–3.83)
Crude OR (unadjusted)	—	—	1.31 (1.02–1.68)
Mantel–Haenszel OR (cytology-adjusted)	—	—	1.44 (1.05–1.98)

CI: confidence interval; OR: odds ratio. Stratum-specific ORs calculated as (a × d)/(b × c) with 95% CI derived from log-transformed standard error. Mantel–Haenszel pooled OR with Robins–Breslow–Greenland variance estimate. Both crude and adjusted ORs exceed 1 with confidence intervals excluding unity, indicating that HPV18 is associated with higher CIN3+ risk than HPV16 independent of cytology distribution.

**Table 6:** Relative Risk (RR) and Odds Ratios (OR) for Selected Comparisons.

Comparison	G1 CIN3+	G1 CIN3−	G2 CIN3+	G2 CIN3−	RR	RR 95% CI	OR	OR 95% CI
HSIL vs. ASC-US	79	27	17	151	7.37	(4.63–11.72)	25.99	(13.36–50.54)
ASC-H vs. ASC-US	122	88	17	151	5.74	(3.61–9.14)	12.31	(6.95–21.81)
HPV16+ vs. HPV Negative	153	899	1	9	1.45	(0.23–9.39)	1.53	(0.19–12.18)
HPV18+ vs. HPV Negative	147	658	1	9	1.83	(0.28–11.79)	2.01	(0.25–15.99)

G1: index group; G2: reference group; RR: relative risk; OR: odds ratio; CI: confidence interval. All estimates reported to two decimal places.

**Table 7:** Univariate Logistic Regression: Candidate Predictors of CIN3+ (n = 1932).

Variable	Crude OR	95% CI	*p*-Value
Cytology: HSIL (ref: ASC-US)	25.99	(13.36–50.54)	<0.001
Cytology: ASC-H (ref: ASC-US)	12.31	(6.95–21.81)	<0.001
Cytology: LSIL (ref: ASC-US)	2.40	(1.17–4.92)	0.016
Cytology: AGC (ref: ASC-US)	3.13	(0.83–11.76)	0.091
HPV16+ with HSIL or ASC-H	19.64	(11.32–34.08)	<0.001
HPV18+ with HSIL or ASC-H	23.83	(13.42–42.34)	<0.001
HPV16+ alone (ref: HPV Negative)	1.53	(0.19–12.18)	0.448
HPV18+ alone (ref: HPV Negative)	2.01	(0.25–15.99)	0.524
Irregular screening (ref: regular)	1.43	(1.12–1.83)	0.004
Age ≥ 50 years (ref: 30–39)	1.18	(0.94–1.49)	0.154
BMI ≥ 30 (ref: normal)	1.09	(0.85–1.40)	0.497
Parity ≥ 2 (ref: nulliparous)	1.11	(0.82–1.50)	0.497

OR: odds ratio; CI: confidence interval. Variables with *p* < 0.10 on univariate analysis were entered into the multivariable model. OR estimates for HPV16/18 alone vs. HPV Negative are statistically unstable (HPV Negative n = 10) and are reported for completeness only.

**Table 8:** Multivariable Logistic Regression: Independent Predictors of CIN3+.

Variable	Crude OR	Crude 95% CI	Adjusted OR	Adjusted 95% CI	*p*-Value
Cytology: HSIL (ref: ASC-US)	25.99	(13.36–50.54)	22.41	(11.28–44.52)	<0.001
Cytology: ASC-H (ref: ASC-US)	12.31	(6.95–21.81)	10.87	(6.08–19.44)	<0.001
Cytology: LSIL (ref: ASC-US)	2.40	(1.17–4.92)	2.18	(1.05–4.52)	0.036
Cytology: AGC (ref: ASC-US)	3.13	(0.83–11.76)	2.89	(0.74–11.27)	0.126
HPV16+ with HSIL/ASC-H	19.64	(11.32–34.08)	17.88	(10.21–31.34)	<0.001
HPV18+ with HSIL/ASC-H	23.83	(13.42–42.34)	21.67	(12.11–38.78)	<0.001
HPV16+ alone (ref: HPV Negative)	1.53	(0.19–12.18)	1.47	(0.18–11.90)	0.718
HPV18+ alone (ref: HPV Negative)	2.01	(0.25–15.99)	1.96	(0.24–15.78)	0.524
Irregular screening (ref: regular)	1.43	(1.12–1.83)	1.38	(1.07–1.77)	0.013
Age ≥ 50 years (ref: 30–39)	1.18	(0.94–1.49)	1.14	(0.90–1.44)	0.281
Model fit: Nagelkerke R^2^ = 0.41; Hosmer–Lemeshow χ^2^ = 6.83, *p* = 0.557					

ASC-H: atypical squamous cells, cannot exclude HSIL; ASC-US: atypical squamous cells of undetermined significance; CI: confidence interval; HSIL: high-grade squamous intraepithelial lesion; HPV: human papillomavirus; OR: odds ratio. Reference categories: cytology ASC-US; HPV Negative. HPV genotype was entered as two binary variables: (1) HPV16/18 combined with HSIL or ASC-H, and (2) HPV16/18 alone (reference: HPV Negative, n = 10). Nagelkerke R^2^ = 0.41; Hosmer–Lemeshow χ^2^ = 6.83, *p* = 0.557 (8 df). OR estimates for HPV16/18 alone vs. HPV Negative are statistically unstable (reference group n = 10) and should not be used for clinical inference.

**Table 9:** Retrospective Estimate of Potential Impact of Risk-Based Triage Strategy on Colposcopy Utilization in the Biopsied Cohort.

Strategy	Total Women Undergoing Colposcopy (n)	Women Undergoing Biopsy (n)	CIN3+ Detected (n)	Potential Reduction in Colposcopy (%)
Current strategy	2748	1932	308	—
Risk-based strategy *	1932	1932	308	29.7%

* Risk-based strategy assumes deferral of immediate colposcopy in women meeting low-risk criteria (normal colposcopy findings, cytology < HSIL, and absence of HPV16/18), as defined in the study protocol.

## Data Availability

The data presented in this study are available on request from the corresponding author. The data are not publicly available due to privacy and ethical restrictions, as they contain potentially identifiable patient health information.

## Abbreviations

The following abbreviations are used in this manuscript:

AGC	Atypical glandular cells
AIS	Adenocarcinoma in situ
ASC-H	Atypical squamous cells, cannot exclude high-grade squamous intraepithelial lesion
ASCCP	American Society for Colposcopy and Cervical Pathology
ASC-US	Atypical squamous cells of undetermined significance
BMI	Body mass index
CI	Confidence interval
CIN	Cervical intraepithelial neoplasia
CIN3+	CIN grade 3, adenocarcinoma in situ, or invasive carcinoma
HPV	Human papillomavirus
hrHPV	High-risk human papillomavirus
HSIL	High-grade squamous intraepithelial lesion
LSIL	Low-grade squamous intraepithelial lesion
OR	Odds ratio
RR	Relative risk
SD	Standard deviation
